# *Flectamus genua*. Gonarthrosis in the Remains of the Blessed Giuseppe Benedetto Dusmet (Catania, Italy, 19th Century AD)

**DOI:** 10.15388/Amed.2025.32.1.24

**Published:** 2025-02-18

**Authors:** Dario Piombino-Mascali, Fausto Grimaldi, Aldo Liberto, Federica Ministeri, Federico Privitera, Cristoforo Pomara

**Affiliations:** 1 Department of Anatomy, Histology and Anthropology, Vilnius University, Vilnius, Lithuania; 2 Pontifical University of Saint Thomas Aquinas – Angelicum, Rome, Italy; 3 Institute of Legal Medicine, University of Catania, Catania, Italy

**Keywords:** relics, osteoarthritis, anthropology, paleopathology, Sicily, relikvijos, osteoartritas, antropologija, paleopatologija, Sicilija

## Abstract

**Introduction:**

This study investigates the bioanthropological and paleopathological features of the late Blessed Giuseppe Benedetto Dusmet, a revered 19^th^-century archbishop of Catania. Dusmet’s remains were examined during the most recent canonical recognition in 2021, providing an opportunity to study the skeletal characteristics that may reflect his lifestyle and health.

**Materials and methods:**

Paleopathological analysis focused on degenerative changes using macroscopic inspection to identify osteoarthritic conditions. Historical records were also consulted to understand the potential connection between his devout religious practices and his physical health.

**Results:**

Significant degenerative and osteoarthritic changes were observed, particularly in the knees. These changes are hypothesized to be linked to Dusmet’s frequent practice of kneeling in prayer, a physical activity historically associated with knee osteoarthritis.

**Conclusion:**

This study highlights how bioanthropological and paleopathological analysis can provide insights into the health and lifestyle of historical figures. The observed knee osteoarthritis in the Blessed Giuseppe Benedetto Dusmet’s remains suggests a possible link between his behavior and the development of joint degeneration. This research adds to our understanding of the physical impact of religious practices and contributes to the study of health in historical figures.

## Abstract (it)

**Įvadas:**

Šiame tyrime nagrinėjamos mirusio palaimintojo Juozapo Benedikto Dusmeto, garbingo XIX a. Katanijos arkivyskupo, bioantropologinės ir paleopatologinės skeletuotų palaikų savybės. Arkivyskupo palaikai buvo atidengti per paskutinį kanoninį pripažinimą 2021 m., todėl atsirado galimybė ištirti jų ypatybes bei patologijas, galinčias atspindėti jo gyvenimo būdą bei sveikatą.

**Medžiagos ir metodai:**

Paleopatologinė analizė buvo atlikta analizuojant degeneracinius sąnarių pakitimus ir vykdant makroskopinę apžiūrą osteoartrito būklei nustatyti. Taip pat buvo susipažinta su istoriniais šaltiniais, siekiant suprasti ryšį tarp jo pamaldžios religinės praktikos ir fizinės sveikatos.

**Rezultatai:**

Pastebėti reikšmingi degeneraciniai ir osteoartritiniai pokyčiai, ypač kelių srityje. Spėjama, kad šie pokyčiai susiję su dažna arkivyskupo maldos praktika klūpint, t. y. fizine veikla, kuri istoriškai gali būti siejama su kelių osteoartritu.

**Išvada:**

Šis tyrimas atskleidžia, kaip bioantropologinė ir paleopatologinė analizė gali padėti suprasti istorinių asmenybių sveikatą bei gyvenimo būdą. Palaimintojo Juozapo Benedikto Dusmeto palaikuose pastebėtas kelio osteoartritas leidžia daryti prielaidą apie galimą ryšį tarp jo elgsenos ir sąnarių degeneracijos išsivystymo. Šis tyrimas papildo mūsų supratimą apie religinės praktikos fizinį poveikį ir prisideda prie žinomų istorinių asmenybių sveikatos tyrimų.

## Introduction

Religious figures who have left a lasting impact through their virtues and deeds are often honored after death in churches and monasteries across Italy [1]. The veneration of such bodies is deeply rooted in the Catholic tradition, and it reflects the cult of relics that began in the early Christian period, particularly during the persecutions of the first few centuries of the Common Era [2]. The Middle Ages marked the peak of relic worship, transforming relics into powerful symbols of status and attracting pilgrims to cities that housed them. This led to the trade and even forgery of relics, a practice that was curbed following the Council of Trent (1545–1563), which mandated that relics must be authenticated by supporting documents [2].

While the spontaneous preservation of religious bodies can occur naturally through favorable burial conditions that promote dehydration [1], the Middle Ages saw anthropogenic methods used to preserve bodies specifically for veneration. These procedures ranged from simple external applications of balms to more elaborate treatments, including evisceration, defleshing, and filling the body cavities with aromatic substances [3].

After the Second Vatican Council (1962–1965), relics and holy remains gained recognition not only as religious symbols but also as items of historical significance. This opened the door to scientific studies aimed at respecting the dignity of the remains while ensuring their proper conservation. These studies focus on accurate identification, preservation assessments, restoration of damage, and the investigation of the individual’s life, health, and the cause of death [4–11].

This paper explores the potential causes of degenerative lesions found in the remains of the Blessed Giuseppe Benedetto Dusmet, a revered 19^th^-century archbishop of Catania, during his recent canonical examination [12]. Specifically, it examines the possibility that the osteoarthritic changes observed in his knees were linked to his frequent acts of kneeling during prayer, worship, and liturgical duties. The investigation conducted in late 2021 aimed not only to conserve the remains but also to produce relics for veneration and remembrance [13].

## Materials and methodology

Born in Palermo (Sicily) in 1818 into a noble family, Giuseppe Dusmet entered the Monastery of Saint Martino delle Scale in Monreale in 1833, made his vows in 1840, and was ordained as a priest shortly afterwards. In 1850, he was transferred to Naples, where he was appointed prior of the Abbey of Saints Severino and Sossio. Just two years later, he returned to Sicily as prior of the Abbey of Saint Flavia in Caltanissetta. In 1858, Dusmet was appointed abbot of Saint Nicolò l’Arena in Catania, a role he would abandon when the premises of this monastery were confiscated by the newly created Kingdom of Italy in 1866. From 1861, the Church of Catania was without a leader, which prompted the then-Pontiff Pius IX to appoint Dusmet as archbishop in 1867 [14].

His episcopal ministry in the archdiocese was marked by his obedience to the Church, love for the clergy, and charity for the people, which distinguished him as an especially merciful father. In the twenty-seven years of his episcopate, he expressed his natural disposition to do good for others and immediately gained recognition for his care towards the poor of the city. Pope Leo XIII eventually appointed him cardinal in 1889. Dusmet died aged 75 in 1894, and his death was met with great sorrow throughout all of Sicily [14]. In 1988, he was declared Blessed by Pope John Paul II. His body now rests in the Cathedral Basilica of Saint Agata in Catania, a destination for devotees who address their prayers to the Cardinal in great numbers.

Initially buried in the city’s monumental cemetery, Dusmet’s remains were first exhumed in 1904 to be transferred to the cathedral. They underwent further canonical inspections in 1951 and 1988, when they were eventually enshrined for worship [14].

**Figure 1:**
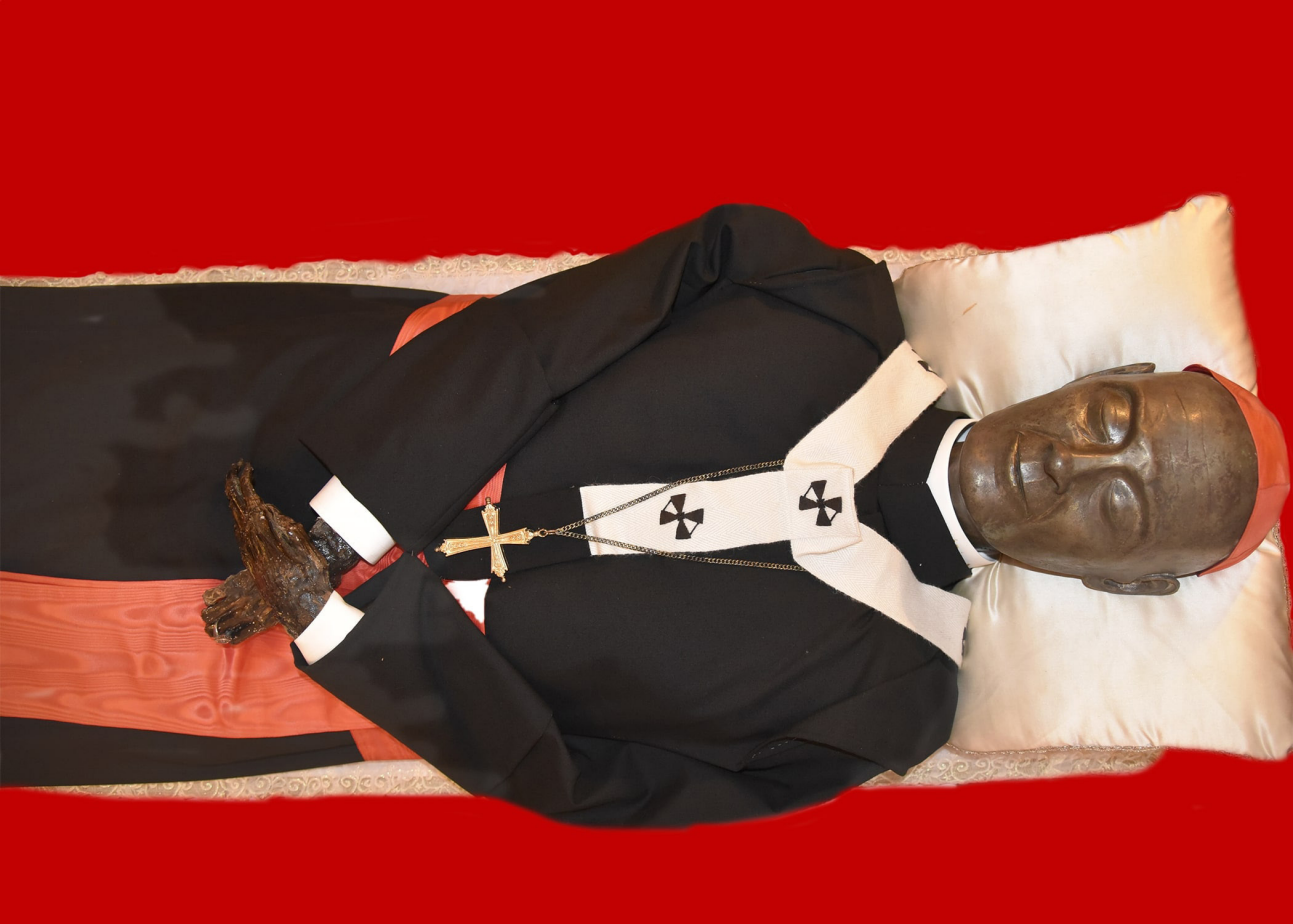
The Blessed Cardinal Giuseppe Benedetto Dusmet during the canonical recognition of 2021

During the latest canonical recognition carried out in late 2021, the authors of this paper were summoned to inspect the remains (Fig. 1), assess their status, and, relying upon standardized procedures, record the Blessed’s bioanthropological and paleopathological features [15]. It should be emphasized that the bones of Dusmet were mounted upon a platform and secured with a clear fishing line, glued with a yellowish stick in different joints, or partly covered by mummified tissue. Thus, in some cases, inspecting the skeletal elements was limited to what could be viewed while the remains were immobilized against the mounting board.

The investigation of Dusmet’s remains relied upon bioarchaeological methods, including sexual assessment of the cranial and pelvic features. Age-at-death estimation was confirmed using multiple indicators, including changes in the pubic symphysis and auricular surface of the ilium, sternal rib ends, and dental and long bone characteristics [16–18]. The remains were also examined for pathological alterations, and scored based on a dedicated codebook, particularly noting degenerative lesions [19–22]. In addition, the stature was estimated using the long bone measurement of the femur, applying the formula for white males [23].

## Results

### Bioanthropological features

The skeletal remains were found neatly arranged in a supine position, with the residue of brown mummified soft tissues located on both scapulae, the right arm, the left hand, and the thoracic spine [24]. Some reddish hair was still attached to the skull. The skeletal system appeared almost complete, except for the hyoid bone, coccyx, tarsals, and two vertebral elements which had been used to create relics or were lost over time. However, nine tarsal or metatarsal elements were kept separately in as many glass containers.

Several features of the skull, including the glabella, the mastoid processes, the occipital protuberance, the chin, and the mandibular angle, together with pelvic traits such as the pubic symphysis, the subpubic angle, the greater sciatic notch, and the overall morphology of the coxae and sacrum clearly indicate the male sex [25]. Regarding the age-at-death estimation, the most reliable indicators, such as the pubic symphysis and the auricular surface of the ilium, were covered with the aforementioned yellowish glue, making a thorough assessment impossible [17,18]. Nevertheless, some segments could be observed, and suggested an advanced age (i.e., 50+). Additionally, the sternal ends of the ribs could be inspected, and estimations were consistent with the findings from the pelvis [16]. Stature estimation, obtained through the femoral maximum length, corresponded to 178.506 ± 3.27 centimeters [23].

### Paleopathological features

During the inspection of the skull, a slight deviation of the nasal septum was observed. The dental apparatus revealed the presence of all maxillary teeth except those spanning the first right premolar – third right molar, which, in the light of the remodeled alveolar plane, had been lost during life. In the mandible, there were four prosthetic incisors linked to the canines through golden wire to the second right premolar, the first right molar (affected by destructive caries), and the third right molar. With the exception of the first premolars on both sides, the other teeth also appeared to be lost antemortem, with subsequent remodeling of the alveoli [19]. The dental wear appeared mild, despite the advanced age of the subject [26].

**Figure 2:**
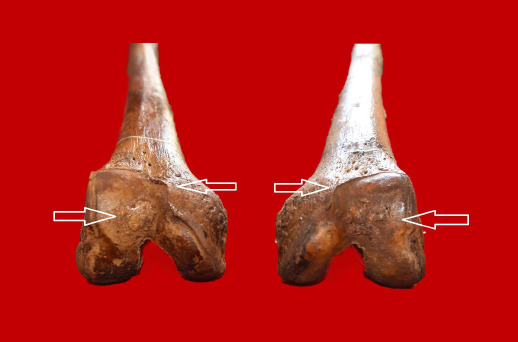
Osteoarthritis of the distal epiphysis of both femora, showing lipping, pitting, and new bone production (arrows)

**Figure 3:**
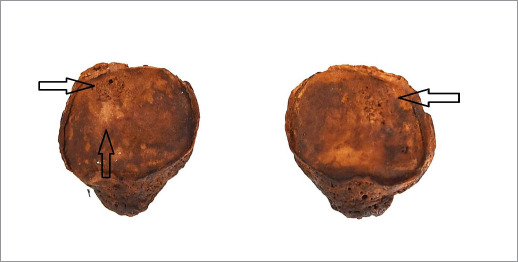
Osteoarthritis is seen on the articular surface of both patellae in the form of lipping, pitting, and slight eburnation (arrows)

The cervical, thoracic, and lumbar tracts of the axial skeleton showed signs of a degenerative joint disease (spondylosis). This was observed even in the sacrum, with the presence of altered articular margins and various exuberant osteophytic beaks (level 3 of severity) [21]. A congenital anomaly was also recorded on the inner surface of the sternum. The appendicular skeleton presented with a slight marginal lipping of the proximal epiphysis of the left humerus, while the right humeral head revealed a slight porosity (level 2 of severity). The lower extremities showed lipping of the articular contours, porosity, and new bone formation on the distal epiphysis of the femur (level 3 of severity) (see Fig. 2). These features were particularly evident at the level of both patellae, where a reduced area of eburnation was also identified on the right side (see Fig. 3). In addition, a trait known as Poirier’s facet was present on the anterior surface of the neck of both femora, both tibiae revealed a squatting facet, while the iliac crests of the pelvic bone revealed entheseal ossification [27].

## Discussion

Osteoarthritis is a chronic and progressive degenerative pathological condition characterized by the loss of cartilage and subsequent lesions resulting from interosseous contact. It is the most common form of synovial joint pathology and can be detected as early as the fourth decade of life. It progresses with age and has a female-sex predilection [19; 20]. This disease is commonly classified as primary, in which no cause is evident, or secondary, when the joint is altered by some other disease or event. Precipitants of the condition include age, genetics, sex, ethnicity, obesity, trauma, and movement [21]. Assessing osteoarthritis requires the observation of at least two features such as marginal osteophytes, new bone formation at the joint surface, pitting, alteration of the articular contours, or eburnation. The latter is a polished, ivory-like area of the subchondral bone, which occurs due to bone-on-bone contact at locations affected by advanced cartilage erosion, which is considered pathognomonic [21].

In this study, we focused on the patellae and femora, which revealed clear evidence of knee osteoarthritis, also known as gonarthrosis [28]. Diagnosis of the condition was based on visual identification via the presence of slight eburnation on the articular surface of the right patella, accompanied by marginal lipping, porosity, and new bone production seen at both the patellar and femoral distal epiphyseal level. This is comparatively more severe than the minor degenerative changes observed in the upper extremities.

Reconstructing precisely the cause of osteoarthritis in the Blessed’s lower extremities without a clinical approach may be difficult, due to the lack of antemortem medical records or the ability to perform an anamnesis. Therefore, the only feasible approach was to use both the skeletal and textual information available, and to narrow down the scale of any possible determinants.

First, the advanced age at death aligns with joint degeneration visible on the spine, notably in the form of exuberant osteophytes and contour alteration, as well as lipping seen on the long bone epiphyses. Although this is a separate phenomenon, it is often associated with osteoarthritis.

Secondly, the potential obesity of the patient was ruled out based on the evaluation of different photographs taken throughout his life, which revealed an average build [28]. Thirdly, no evidence of trauma hypothetically related to osteoarthritis formation was recognized in the areas affected. As a conclusion, the most likely explanation for the relatively more severe degenerative arthropathy visible on both knee joints may be sought in individual behavior [29].

It can reasonably be argued that physical activities, such as kneeling and squatting, can cause or exacerbate knee osteoarthritis [30]. In this specific case, historical data shed light on a possible etiology related to the religious practices Dusmet engaged in throughout his more than 50 years of service. Genuflection [Latin: *genu flectere*, to bend a knee] expresses an attitude or posture during private praying, and a gesture of reverence. Kneeling while praying is common among Christians, particularly in the Roman Rite, and involves bending one or both knees to touch the ground. The liturgical rules regarding kneeling apply to both clergy and laypeople. Kneeling is required during adoration of the unveiled Blessed Sacrament (both knees), when doing reverence to the Blessed Sacrament enclosed in the tabernacle (right knee only), and on Good Friday until Holy Saturday, when passing before the unveiled cross upon the high altar [31].

Interestingly, Dusmet’s first biography states that the clergyman was often seen kneeling while praying at night in the chapel of his archbishopric [32]. Furthermore, other potential indicators observed on the skeleton, such as the gluteus maximus and piriformis prominent insertions, may indicate leg extension due to rising from a kneeling or genuflecting position [33]. However, it should be mentioned that exuberant entheseal changes are commonly seen in elderly individuals, and therefore pose a challenge in terms of interpretation [34]. Poirier’s femoral facets and tibial ‘squatting’ facets have also been reported to result from extension and flexion [27], potentially reflecting motion in this instance, adding to the overall picture. Combining the osteological evidence collected with written sources, a circumstantial case can thus be made, hypothesizing that the disease affecting the late Cardinal’s knees, more pronounced on the right side, was linked to frequent bending for religious purposes. This would suggest an occupational etiology for the lesions observed rather than a simple feature of the aging process.

## Conclusion

The anthropological and paleopathological inspection of the remains of Cardinal Dusmet revealed evidence of marginal spondylosis of the spine and joint degeneration of the long bone epiphyses, as well as knee osteoarthritis (gonarthrosis) which had not been observed during previous examinations. The latter condition in particular is consistent with both historical sources and the actions regularly performed by the clergy for worship and liturgy, as dictated by the religious rule. Identified cases like this one serve as a unique source of information, not only for outlining a biological profile but also for suggesting or confirming the habits and occupations these individuals had during their lifetime.
